# The need for vigilance: melioidosis mimicking tuberculosis

**DOI:** 10.1128/asmcr.00018-25

**Published:** 2025-07-31

**Authors:** Sushree Sarathi, Pragya Agarwala, Padma Das, Vinay R. Pandit

**Affiliations:** 1 Department of Microbiology, All India Institute of Medical Sciences 417408, Raipur, Chhattisgarh, India; 2 Department of General Medicine, All India Institute of Medical Sciences, Raipur, Chhattisgarh, India; Vanderbilt University Medical Center, Nashville, Tennessee, USA

**Keywords:** melioidosis, *Burkholderia pseudomallei*, lymphadenitis, neck abscess

## Abstract

**Background:**

Melioidosis, caused by *Burkholderia pseudomallei*, is often misdiagnosed due to its clinical similarity with tuberculosis and broad-spectrum clinical manifestations. The infection is particularly prevalent in endemic areas, and a high index of suspicion is required for timely diagnosis and treatment.

**Case Summary:**

We report a 50-year-old male construction worker from central India initially diagnosed with presumptive smear-negative tubercular lymphadenitis. Despite anti-tuberculosis treatment, his condition worsened, prompting further investigation. Culture and biochemical tests identified *B. pseudomallei*, confirming melioidosis. The patient was started on intravenous ceftazidime and later transitioned to oral cotrimoxazole. He showed significant clinical improvement after treatment.

**Conclusion:**

This case underscores the importance of considering melioidosis in the differential diagnosis of patients with lymphadenitis, particularly in areas where the pathogen is endemic. Timely identification and appropriate treatment are crucial for effective management. Heightened awareness of melioidosis is essential to avoid misdiagnosis and ensure optimal outcomes for patients.

## INTRODUCTION

Melioidosis, a clinical condition in humans and animals, is the result of an infection with *Burkholderia pseudomallei*, a non-fermenting, Gram-negative, oxidase-positive, motile, aerobic bacillus that lives in damp soil, water, and plants in local regions and is endemic in Southeast Asia and regions of northern Australia (1, 2). The disease in humans arises after the bacterium enters the body through contact with defective skin with soil or water, inhalation, and ingestion. During the rainy season, many cases are documented. Melioidosis is frequently misdiagnosed because its diverse clinical signs and symptoms can be mistaken for those of other illnesses, such as tuberculosis, sepsis/septic shock, and community-acquired pneumonia (CAP) (3). The clinical symptoms range from a short febrile illness and localized abscess to fatal septicemia (4). It is frequently observed in alcoholics and diabetics (5). *B. pseudomallei* seldom causes suppurative lymphadenitis. Only a small number of case reports with melioidosis presenting as lymphadenitis are described in the medical literature (6, 7). In laboratories with poor resources and without automated identification systems, it is frequently reported as *Pseudomonas* or a non-fermenting Gram-negative bacillus. Diversities in clinical presentations and difficulty in manual identification lead to underreporting

## CASE PRESENTATION

**Fig 1:**
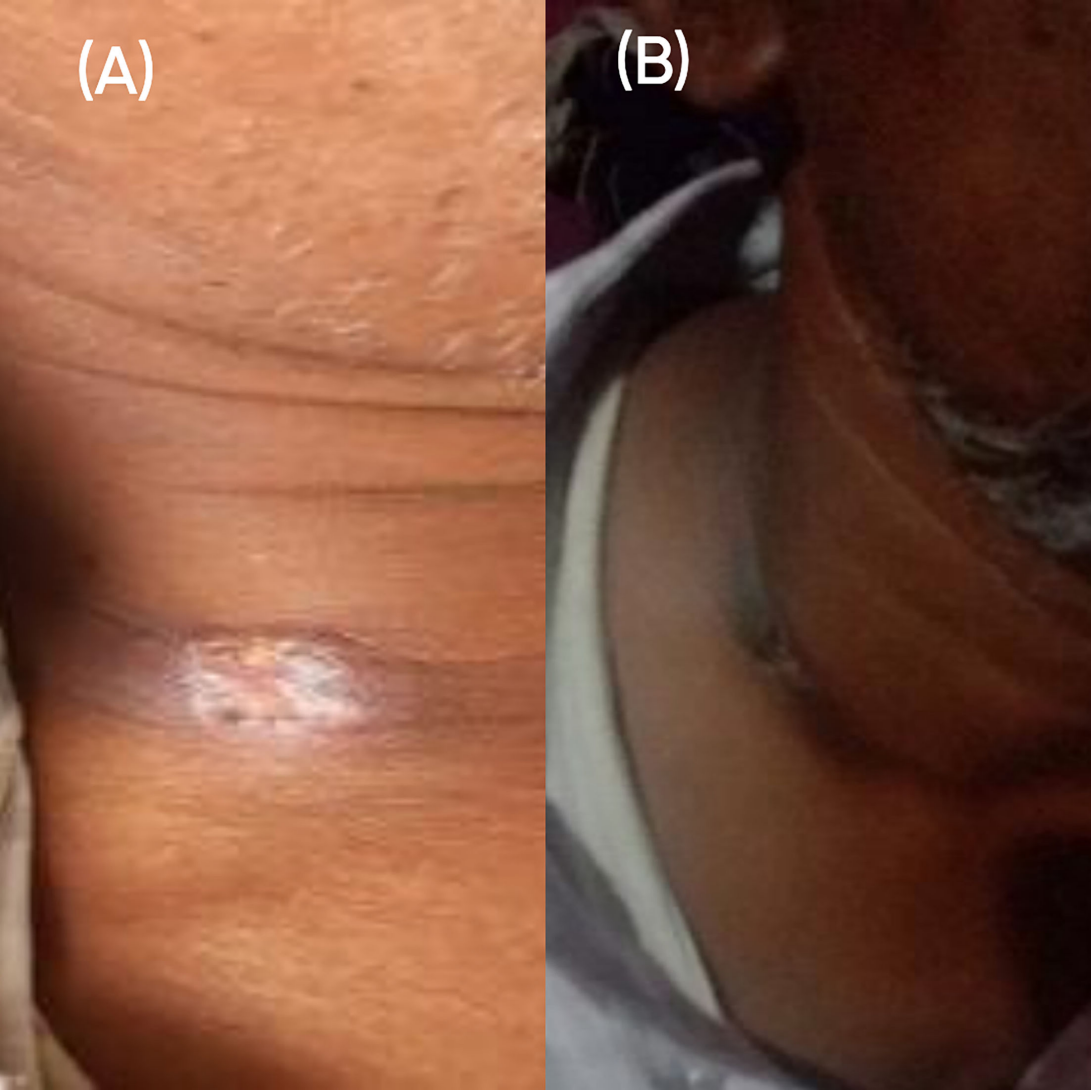
Clinical picture showing the swelling (**A**) before treatment and (**B**) after treatment.

A 50-year-old male construction worker was referred to our center with a provisional diagnosis of presumptive smear-negative tubercular lymphadenitis in April 2023. He had presented at a local hospital around 2 months prior with a complaint of low-grade intermittent fever for 20 days, which was acute in onset, associated with chills and rigor, and subsiding with antipyretics. He noticed a gradually increasing swelling over the right side of the neck 10 days after the onset of the fever. The development of the swelling was insidious and was associated with throbbing pain and redness. There was no local rise in temperature. After 3 days, the swelling developed an opening and started discharging pus. On local examination, the swelling was soft to firm in consistency, measuring 3 × 2 cm, with central ulceration and associated purulent discharge (Fig. 1A). A general physical examination confirmed painful right supraclavicular cervical lymphadenopathy without any association with local pathology in the ear, nose, throat, and oral cavity.

**Fig 2:**
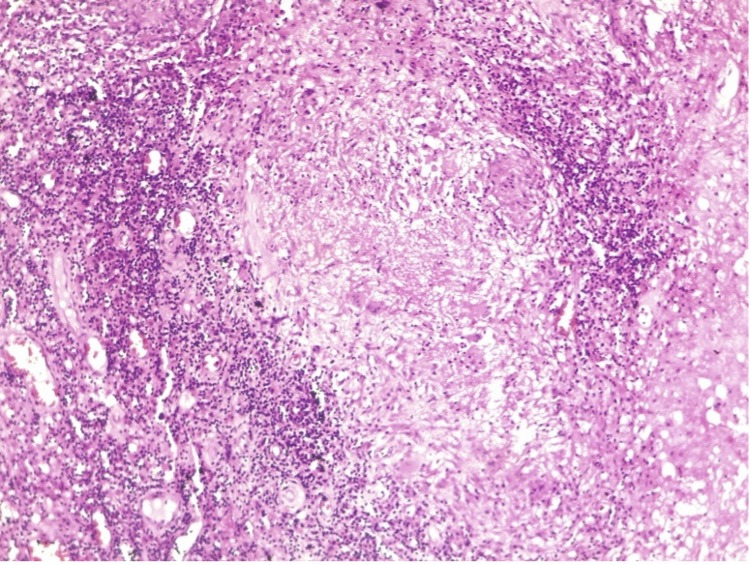
FNAC showing necrotizing granulomatous picture (Giemsa, ×10).

Fine needle aspiration cytology (FNAC) of the lymph node was done and sent for Ziehl-Neelsen (ZN) staining, Cartridge-Based Nucleic Acid Amplification Test (CBNAAT), and histopathological examination (HPE) at a local hospital. ZN staining and CBNAAT of the FNAC sample were negative for *Mycobacterium tuberculosis*. However, histopathological evidence of necrotizing granulomatous lesion with lymphocytic infiltration (Fig. 2) suggested it as a case of presumptive smear-negative tubercular lymphadenitis with diabetes mellitus based on history and recent laboratory reports. Routine aerobic bacterial cultures were not conducted at this stage. This reflects a diagnostic bias toward tuberculosis, likely influenced by its high prevalence in the region, involvement of cervical lymph nodes, and presence of necrotizing granuloma in HPE. Antitubercular treatment (ATT) and insulin were started, but even after a month of rigorous TB treatment (isoniazid, rifampicin, pyrazinamide, and ethambutol fixed dose combination of 3 tablets daily), his symptoms worsened, and he remained feverish, necessitating a referral to our facility, the sequence of events of which is described below.

**Fig 3:**
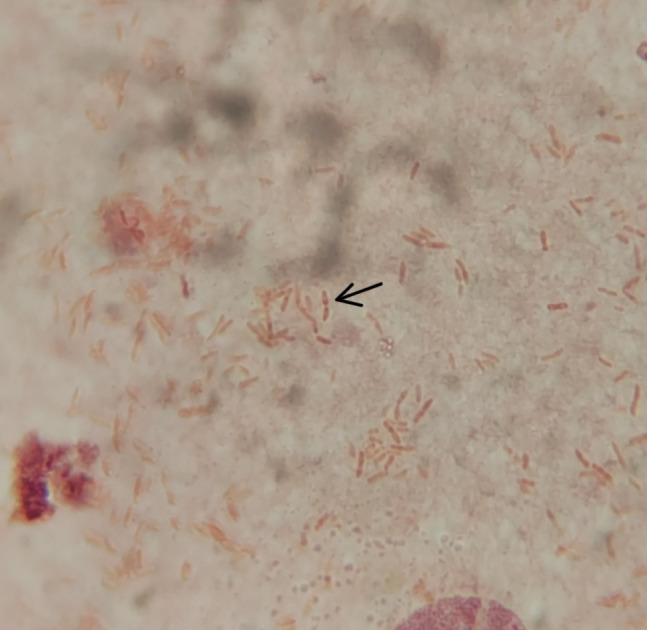
Gram’s stain – bipolar staining (Gram’s, ×1,000).

**Fig 4:**
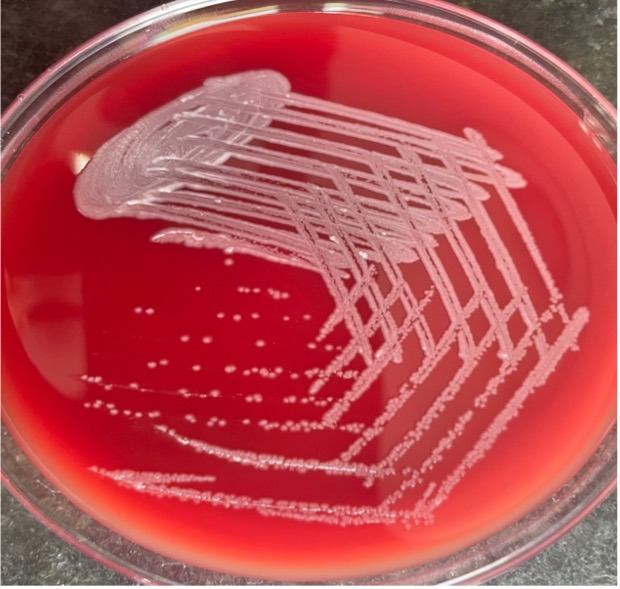
Colony morphology on blood agar after 24 h of incubation.

The diagnostic workup continued at our center to evaluate new possibilities for diagnosis. The attending clinician advised a bacterial culture, sensitivity, and ZN-staining of the aspirate from the lesion. Gram stain revealed numerous leukocytes, with few Gram-negative bacilli having characteristic safety pin (bipolar staining) appearance (Fig. 3). ZN-staining showed no acid-fast bacilli. After inoculation on both blood and MacConkey agar, the plates were incubated overnight at 37°C. Initial laboratory findings revealed the total leukocyte count to be 10.33 × 10^3^ /μL, erythrocyte sedimentation rate 120 mm/h, a normal differential WBC count along with marginally increased HbA1c (6.7%) and post-prandial blood sugar (129 mg/dL). The chest X-ray findings were normal. After 24 h of incubation, large, creamy, and smooth non-hemolytic colonies developed on blood agar,(Fig. 4) which was non-lactose fermenting, and had a metallic sheen on MacConkey agar. The organism was motile, oxidase, and catalase positive. Further characterization and identification were done by various biochemical tests and an automated identification system.

Biochemical tests revealed that it produced arginine dihydrolase and reduced nitrate to nitrite. It could utilize glucose and maltose oxidatively and hydrolyzed urea, but citrate was not utilized, and the organism could grow at 42°C. Interestingly, the colony became dry and wrinkled after 48 h. Vitek 2 identification system identified the organism as *Burkholderia pseudomallei* with 99% probability. The antibiotic susceptibility was done according to the disk-diffusion breakpoints mentioned in EUCAST. The organism was resistant to aminoglycosides, amoxicillin-clavulanate, and sensitive to ceftazidime, meropenem, and cotrimoxazole. Paired blood cultures were advised to rule out generalized bacteremia. The neck swelling showed progressive enlargement over the subsequent week, accompanied by increased tenderness and discharge, despite ongoing ATT. In the follow-up consultation after 3 days, the diagnosis was revised as melioidosis with newly diagnosed diabetic status. The ATT was discontinued. The intensive phase of treatment was started with an intravenous infusion of ceftazidime 2 g QID over 2 h for 14 days, according to the 2020 Revised Darwin melioidosis guideline, along with insulin therapy (8). Eleven days post-treatment, the patient became afebrile. Shortly after, his swelling began to reduce in size.

The patient was discharged on eradication therapy with oral cotrimoxazole (960 mg once daily) for 3 months with in-between follow-ups. During the subsequent revisits, the lesion completely subsided without any recurrence (Fig. 1B).

## DISCUSSION

Melioidotic abscesses involving the head and neck region have been reported in several endemic countries, including Malaysia, Indonesia, Taiwan, and India (9). However, melioidosis-related suppurative lymphadenitis that manifests as neck masses or abscesses in the head and neck region has been reported scarcely in the literature. In non-endemic areas like the USA or the UK, melioidotic neck masses have typically been reported in populations of non-indigenous immigrants or travelers (10).

In a study done in Thailand, 23% (11/49) of the culture-positive melioidosis cases presented as suppurative lymphadenitis (11). According to another review, 40% of cases of localized melioidosis worldwide are associated with cervical lymphadenopathy (12).

Cases of melioidosis in India are mainly from South India and sporadically reported from eastern and western parts of the country. However, there are occasional reports from coastal states. So, there is a high possibility that cases are neither diagnosed nor reported from some tropical areas of the country. Moreover, not all diagnosed cases are published. Hence, the number of actual cases is much higher than realized. While the number of reported cases is increasing, data on the precise regional distribution of melioidosis, particularly in central India, are still limited. Surveillance studies and hospital-based research are needed to understand the true burden of the disease better and identify high-risk areas.

Multiple reports from India have shown cases of melioidosis resembling and co-infecting tubercular cold abscesses (10). Therefore, especially in a tuberculosis-endemic nation like India, melioidosis should be included in the differential diagnosis of suppurative or granulomatous regional lymphadenitis in the neck region in addition to tuberculosis.

The clinical presentation of melioidosis can vary depending on the route of infection, with cervical adenitis often resulting from inhalation or ingestion, possibly via exposure to contaminated water sources, and inguinal adenitis typically occurring from traumatic inoculation. In one study by Mohan et al. (2021) in the pediatric population, 95% of melioidosis cases in the head and neck region presented with cervical lymphadenitis, which was thought to be related to the acquisition of *B. pseudomallei* through the oral/nasal mucosa or conjunctiva (13).

The present report describes a smear and culture-positive case of melioidotic cervical lymphadenitis in a tertiary care center in central India.

In our study, since the patient worked at construction sites, and the organism can be found in soil and surface water, the infection may have been contracted by inhalation or traumatic bacterial inoculation. A high level of suspicion and a thorough search aided in establishing the diagnosis, allowing for the initiation of appropriate therapy.

### Conclusion

This report underscores the importance of *Burkholderia pseudomallei*, an entity that is currently under-recognized as a cause of cervical lymphadenitis, mimicking tubercular lymphadenitis. In a country like India, which is endemic for both melioidosis and tuberculosis, it is pertinent to identify the infecting agent unmistakably, keeping in view the similar presentation and the entirely different treatment of both. This case also highlights the consequences of incomplete initial microbiological evaluation. The absence of routine aerobic cultures during the initial workup led to a diagnostic delay and inappropriate empirical therapy for tuberculosis. In TB-endemic settings, while tuberculosis may be a common cause of lymphadenitis, exclusive focus on it can lead to “diagnostic fixation.” A comprehensive microbiological assessment is of utmost importance to avoid misdiagnosis
